# Aerosol Emissions from Heated Tobacco Products: A Review Focusing on Carbonyls, Analytical Methods, and Experimental Quality

**DOI:** 10.3390/toxics11120947

**Published:** 2023-11-21

**Authors:** Roberto A. Sussman, Federica Sipala, Rosalia Emma, Simone Ronsisvalle

**Affiliations:** 1 Institute of Physical Sciences, National Autonomous University of Mexico UNAM, Mexico City 04510, Mexico; 2 Department of Drug and Health Sciences, University of Catania, 95123 Catania, Italy; 3 Center of Excellence for the Acceleration of Harm Reduction (CoEHAR), 95123 Catania, Italy; 4 Department of Clinical and Experimental Medicine, University of Catania, 95123 Catania, Italy

**Keywords:** heated tobacco products, carbonyls, aerosols, analytical methods

## Abstract

We provide an extensive review of 17 independent and industry-funded studies targeting carbonyls in aerosol emissions of Heated Tobacco Products (HTPs), focusing on quality criteria based on the reproducibility of experiments, appropriate analytic methods, and puffing regimes. Most revised studies complied with these requirements, but some were unreproducible, while others failed to consider analytical variables that may have affected the results and/or produced unrealistic comparisons. We also provide a review of the literature on the physicochemical properties of heated tobacco and HTP aerosols, as well as the evaluation of HTPs by regulatory agencies, addressing various critiques of their relative safety profile. The outcomes from the revised studies and regulatory evaluations tend to agree with and converge to a general consensus that HTP aerosols expose users to significantly lower levels of toxicity than tobacco smoke.

## 1. Introduction

The smoke emitted by conventional tobacco cigarettes (CCs) is an extremely toxic nicotine delivery mechanism. As a consequence, cigarette smoking is responsible for over 7 million premature deaths each year, including non-smokers exposed to secondhand smoke [1,2]. In spite of a persistent institutional effort to address this global health problem, over 1200 million people continue smoking, mostly in lower- and middle-income countries. Therefore, as a complementary strategy to reinforce traditional smoking cessation and prevention methods, an alternative approach to address the harms of smoking comes from a Tobacco Harm Reduction (THR) perspective [3,4,5,6], based on substituting the toxic nicotine delivery from CCs by consumer products whose nicotine delivery does not involve combustion. While THR products are not risk-free, they expose users to a significantly lesser content of hazardous and potentially hazardous compounds (HPHCs)

Among the THR products, Electronic Cigarettes (ECs) and Heated Tobacco Products (HTPs) are generically known as Electronic Nicotine Delivery Systems (ENDS) [7], as they replace the highly toxic nicotine delivery through tobacco smoke via delivery through electronically generated aerosols not produced by combustion. The plausibility of the THR approach through the significant reduction in exposure to HPHCs in ENDS users has a solid physicochemical and empirical basis (see discussion in Section 3). Tobacco smoke (mainstream and sidestream emissions) is a product of the incomplete combustion of tobacco biomass that initiates the ignition of the tobacco leaf at 800–950 °C, a highly energetic exothermal oxidizing process that triggers a series of complex endothermic processes producing over 7000 detected compounds, thousands of these HPHCs in significant concentrations, including around 70 known carcinogens [1]. In contrast, ENDS aerosols are generated by heating processes taking place at temperatures well below 400 °C, the threshold temperature of tobacco ignition (180–270 °C for ECs and 200–350 °C for HTPs). Therefore, as a consequence of this fundamentally decreased physicochemical complexity, the overwhelming majority of compounds (including HPHCs) found in the smoke of CCs are absent in ENDS aerosols, while those present are found in substantially smaller concentrations relative to their concentrations in tobacco smoke.

Although all ENDS share some common features, there are significant differences between them [5,6,7,8]. Both ECs and HTPs generate aerosols from the condensation of supersaturated vapors, but in ECs, it is the vapor produced by heating a liquid solution (the e-liquid), while in HTPs, it is produced by heating suitably treated forms of tobacco. Another important difference is their standardization and manufacture: ECs exhibit an enormous diversity of devices and designs, with a wide range of power settings, e-liquids, adjustable nicotine levels, and flavoring choices. The majority of EC devices are manufactured by small/medium-emerging industries in China, although the tobacco industry is also present in the EC market. In contrast, HTPs are relatively standardized products with far fewer designs than ECs. Also, all HTPs are manufactured by major tobacco industries: Phillip Morris International (PMI), British American Tobacco (BAT), and Japan Tobacco International (JTI) [5,6].

Contemporary commercial HTPs have the following three main designs of aerosol generation on specially prepared tobacco “sticks” [9,10,11,12]:

- eHTP: aerosols are generated by directly heating specially treated tobacco sticks (or “heets”). The gender includes two series of devices, IQOS™ Philip Morris International (PMI) (Stamford, U.S.A.) and glo^TM^, manufactured by and British American Tobacco (BAT) (London, UK);
- aHTP: an independently generated aerosol is filtered through tobacco sticks. There are two devices of this type, eFuse™ (BAT, London, UK) and Ploom™ (JTI, Geneva, Switzerland), respectively, manufactured by BAT and JTI;
- cHTP: a carbon rod is used to heat tobacco sticks. The now obsolete Eclipse™ (BAT, London, UK).

Since the HTP market is overwhelmingly dominated by eHTP devices, we will consider only these products in this review, understanding that (unless specified otherwise) the term “HTP” will denote either one of the eHTP devices: IQOS™ or Tobacco Heating System 2.2 (THS2.2) from PMI and glo™ or Tobacco Heating Product THP1.0 from BAT (we will use, henceforth, only the commercial names “IQOS”, “glo”, and “Ploom”).

In this review, we provide a revision of 17 studies on HTP aerosol emissions (9 industry-funded and 8 independent), focusing mostly on their experimental quality and analytical methods of quantification of carbonyls (especially aldehydes), which are, in general, the most abundant HPHCs found in aerosol emissions of HTPs (and also in ECs). As explained in Section 3, these byproducts are produced by thermal decomposition (low-temperature pyrolysis or torrefaction) of the biomaterial and its solvents, taking place during the heating process of aerosol formation, irrespective of the presence of oxygen or oxidizing agents. Other HPHCs, such as carbon monoxide (CO), polycyclic aromatic hydrocarbons (PAHs), volatile organic compounds (VOCs), phenols, tobacco-specific nitrosamines (TSNAs), and other compounds, are also found as thermal decomposition byproducts in HTP aerosols but are typically at lower levels than aldehydes.

The use of HTPs has become widespread, encompassing many countries, including Japan, South Korea, Italy, Switzerland, Israel, Russia, Germany, the UK, and many urban locations in the EU [5,6,9,10,11,12]. Their introduction and marketing in the US have been slower. In December 2016, PMI submitted to the US Food and Drug Administration (FDA) a Modified Risk Tobacco Product (MRTP) application for the IQOS, with the agency authorizing its marketing in 2020 under an “exposure modification” order [13], though a patent dispute has prevented its entry into the market. However, in Japan, HTP usage (not only IQOS) has become more popular and widespread, with an estimated 10 million users (half of the world usage [5,6]), a 50% drop in CC sales between 2016 and 2023 (from 144.8 billion US dollars to 69.4 billion [6]). Diverse aspects of HTP use in Japan are described and discussed in the Special Issue of the International Journal of Environmental Research and Public Health [14]. In particular, among the studies in [14], it is worth mentioning [15], which examined the specific context of HTP usage and findings from Japanese demographic surveys:

- 18.4% in 2017 used a single tobacco product (78.8% cigarettes and 5.2% HTPs), while 3.2% used multiple tobacco products (with 10.5% using HTPs) [16];
- In 2018, monthly HTP use was 2.7% (1.7% daily use), with 67.8% and 25.0% current and former smokers, respectively, and 1.0% never smokers. IQOS and menthol flavor were used at 64.5% and menthol flavor (of all products) at 41.5%, respectively. IQOS was preferred by younger respondents and Ploom TECH by older respondents and non-daily HTP users [17].

However, the prevalence of HTP usage has increased since 2018, as reflected by the continuing decline in cigarette sales. 

The present review reveals a generalized consensus among industry and independent studies we have revised (also sustained by regulatory agencies) that HTP aerosols contain far fewer and in significantly smaller concentrations of HPHCs than in CC smoke (as recognized by public institutions and regulatory agencies [13,18,19,20]). However, we recognize that the presence of these HPCPs, even at minute levels, is concerning. In particular, aldehydes (especially formaldehyde, acetaldehyde, and acrolein) play a major role in the toxicity and carcinogenesis of tobacco smoke [21]; hence, their presence in HTPs and ECs requires active monitoring and vigilance. The International Agency for Research on Cancer (IARC) classifies formaldehyde as a human carcinogen (Group 1) [22]. Acetaldehyde is possibly carcinogenic to humans (Group 2B), according to the IARC, and acrolein is probably carcinogenic to humans (Group 2A) [23,24]. The full toxicological assessment of the health effects of substituting CCs with HTPs requires further research on the interaction among toxicants, biomarkers, and pre-clinic and clinical studies [21]. However, emission studies showing much lower levels of toxicity in HTPs relative to CCs constitute the basis (and fundamental) finding that justifies undertaking this long research process. 

The section-by-section content is as follows. In Section 2, we provide the PRISMA selection of studies on HTPs to revise and a methodological flow diagram to present graphically the aims and scope of the present review. Section 3 provides an extensive review of important theoretical background material: physicochemical properties of CC smoke, thermophysical and thermochemical processes of heating tobacco without combustion, and characteristics of HTP aerosols. In Section 4 and Section 5, we provide reviews of independent and industry studies of HTP emissions, focusing on carbonyls. The outcomes of this revision are extensively discussed in Section 6, including a summary of analytic methods (Section 6.1), arguments that might challenge the consensus on HTP relative safety with respect to CCs (Section 6.2), and the evaluation of IQOS by the U.S. FDA (Section 6.3), the positioning of the World Health Organization (WHO) (Section 6.4), the speculative unsustainable claim that HTP aerosols can be characterized as “smokes” (Section 6.5), and our evaluation of the reliability of the studies (Section 6.6). Our conclusions are presented in Section 7.

## 2. Materials and Methods

**Figure 1:**
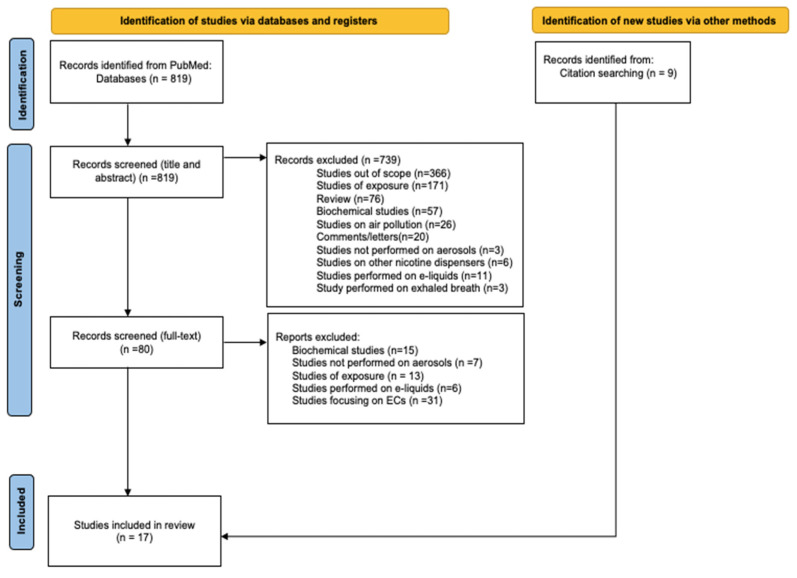
PRISMA-recommended workflow.

We searched the PubMed database for articles on carbonyls in aerosol emissions from CCs and ENDS products following the workflow recommended by PRISMA (Figure 1). The keywords searched were {carbonyl OR aldehyde OR formaldehyde OR acetaldehyde OR acrolein} AND {cigarette aerosol or e-cigarette aerosol or heated tobacco product aerosol}. An initial search was performed on the titles and abstracts of the articles published after 2018. Independent studies and studies funded by tobacco companies reviewed by Simonavicius et al. (2018) [25] and by El-Kaassamani et al. (2022) [26] were also included. Next, a full-text search was performed to exclude articles that did not meet our purpose, such as studies focused solely on ECs, reviews, exposure studies, and studies not focused on carbonyls. We critically analyzed articles that were not excluded to highlight the limitations of analytical methods and puff regimens and capture the protocols used in the analysis of carbonyls from cigarette smoke and HTP aerosols. Specifically, we examined compliance using the following criteria:

- Studies were conducted on aerosols collected according to the standardized recommended puffing protocol of the Cooperation Centre for Scientific Research Relative to Tobacco (CORESTA);
- Aerosols were adequately treated for carbonyl entrapment;
- Analytical methods were adequate and reproducible, with particular attention to blank analyses;
- Samples were stored adequately prior to analysis.

The PRISMA-recommended workflow that was used is displayed in Figure 1.

**Figure 2:**
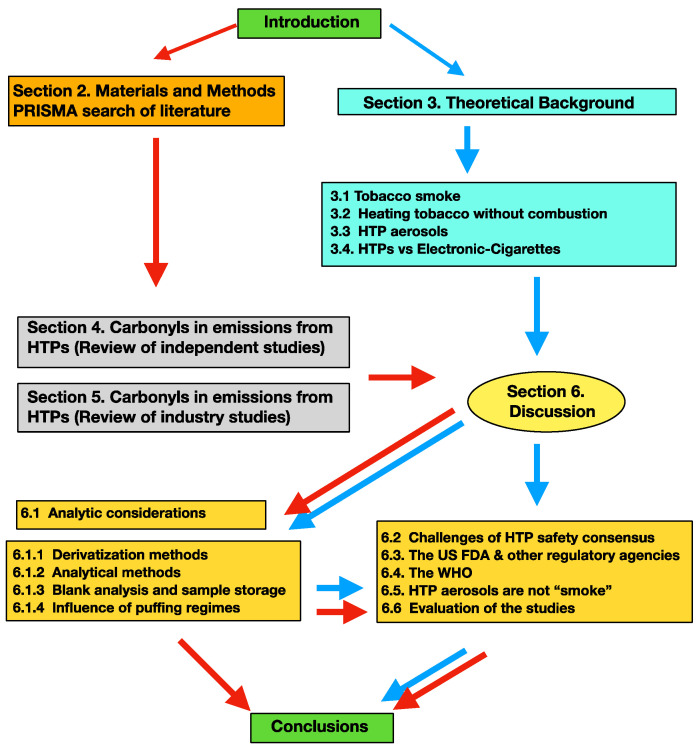
Methodological flow diagram.

In Figure 2, we provide a methodological flow diagram that illustrates the aims and scope of the present review and can be used as a reading guideline. While all the sections and subsections are mutually connected, some readers might prefer looking at some sections first and others afterward. Those who prefer reading first the reviews of the 17 studies might follow the red arrows, while those wishing to read first the theoretical background might follow the blue arrows. 

## 3. Theoretical Background

The present section provides a review of the theoretical and experimental background of the development and operation of HTPs. In particular, we focus on the physicochemical characteristics of tobacco smoke, the thermophysical and thermochemical processes behind heating tobacco, and the characteristics of HTP aerosols.

### 3.1. Tobacco Smoke

The term “tobacco smoke” denotes a highly dynamic set of chemically complex aerosols produced by the incomplete combustion of tobacco biomass when smoking CCs [1,27,28,29]. The particulate phases of these smoke aerosols consist of solid particles and liquid droplets composed of complex hydrocarbon combinations and multiple organic and inorganic compounds, all suspended in gaseous phases composed of equally complex vapor mixtures. These aerosols are initially generated by a highly energetic exothermic and oxidizing ignition process (burning) taking place in the tip of the cigarette, reaching 910–920 °C when the smoker puffs and remains smoldering at 450–800 °C when the cigarette is not puffed. Igniting the initial smoke leads to the formation of three distinct forms of “smoke” aerosols:

- “mainstream emission”: the inhaled smoke from the mouth end of the puffed cigarette, cooled and transported by the cigarette rod and exhaled by the smoker;
- “side stream emission”: the smoke released directly into the environment from the burning/smoldering tip of the cigarette;
- “environmental tobacco smoke” (ETS): the smoke formed by an aging mixture of exhaled mainstream and sidestream emissions released into the environment, which diffuse, dilute, and react with ambient air chemicals.

The sidestream emission accounts for approximately 80% of the combined smoke from the two emissions released into the environment, but most of the particulate phase of ETS (57–85%) comes from the sidestream emission, while most of its gas phase (87–99%) comes from exhaled mainstream smoke [26]. 

While the ignition is oxidizing and exothermic, thus generating an oxygen-depleted (hydrogen-rich) zone close to the burning tip of the cigarette rod, the inhaled mainstream emission is formed by a series of overlapping endothermic (energy-absorbing) physicochemical processes that take place along the cigarette rod during cigarette puffing: distillation and sublimation that initiate a few millimeters below the oxygen-depleted hydrogen-rich ignition zone (the glowing cone), rapidly (milliseconds) cooling the ignition smoke to 200–600 °C and further to ambient temperatures as this smoke is transported along the cigarette rod, with significant oxygen consumption to sustain the whole combustion process, releasing heat and incomplete combustion byproducts, such as carbon and nitrogen oxides (CO, CO_2_, NO_x_). 

Multiple chemical constituents (up to 7000 detected compounds) are produced along the rod by pyrolysis/pyrosynthesis in rapidly cooling distillation/sublimation cycles, all of which supersaturate even less volatile gases, forming mixtures of vapors produced by condensation ultrafine liquid droplets of complex chemical composition that grow by (hygroscopic) water absorption and coagulation, forming the “tobacco aerosol residue” (TAR) retained in the laboratory by filtering out water and nicotine. The gas and particulate phases of mainstream emissions comprise 92% and 8%, respectively, in terms of mass proportion, with the gas phase composition approximately consisting of 58% nitrogen, 12% oxygen, 13% carbon dioxide, 3.5% carbon monoxide, 5% volatile organic compounds (VOCs), 0.5% hydrogen, and 1% water. In terms of chemical species, mainstream emissions contain hundreds of chemical species: aliphatic, acidic, and aromatic hydrocarbons; amines; amides; carboxylic acids; esters; aldehydes; ketones; phenols; nitrosamines; nitriles; ethers; metals; and many others.

Mainstream and sidestream emissions generally have similar chemical compositions, but they are distinct aerosols: mainstream smoke is slightly acidic, and sidestream smoke is slightly alkaline (from a larger proportional presence of ammonia). Sidestream emissions also contain a larger share of solid particles and tend to have a higher proportion of particulate matter with smaller diameters. The sidestream/mainstream ratio of yields depends on the specific chemicals and is highly variable. For example, nicotine in mainstream emissions is exclusively protonated and concentrated in the particulate phase, while it has been reported to be 50–80% in the gaseous phase of sidestream emissions; it has also been reported as almost entirely in the particulate phase [27,28,29].

### 3.2. Heating Tobacco without Combustion

It is important to understand how aerosols can be generated from tobacco materials without burning them. There is abundant literature on this issue, including two documents produced by industry authors (PMI and BAT [30,31]). The incomplete combustion of a biomass initiates along with exothermic oxidizing reactions at (or above) a threshold temperature given by its ignition point [32,33]. However, combustion processes are not activated if the biomass is exothermically heated below its ignition point, leading to thermophysical and thermochemical degradation processes (distillation, evaporation, and low-energy pyrolysis or torrefaction) that give rise to volatile and semi-volatile vapor mixtures determined by specific biomasses. These vapors, when saturated, condense to form aerosols that cannot be categorized as “smokes”, as their particulate phase is made mostly of liquid droplets, and they emerge irrespective of the presence of oxygen or oxidizing agents, all of which represent completely different physicochemical features of smoke aerosols. Aerosols not categorized as smoke can be generated by similar processes when the tobacco mass is heated at its ignition temperature of 400 °C or above.

Tobacco biomass consists of nitrogenous compounds and biopolymers, such as hemicellulose, cellulose, and lignin [34]. When subjected to an exothermic heat supply below the ignition threshold of 400 °C, these constituents undergo thermal degradation processes similar to those of other biomasses: dehydration, volatile release, and thermal degradation from low-energy pyrolysis [35,36,37]. The standard laboratory method to study the heating of tobacco (or any biomass) is Thermogravimetric Analysis (TGA) combined with Fourier Transform Infra-Red (FTIR) spectrometry, which identifies the thermophysical processes by thermogravimetric curves displaying the absolute and differential rates of mass loss in contiguous ranges of increasing temperatures, allowing the quantification of compounds in the produced aerosol by deconvolution techniques applied to the TGA spectrum. 

Untreated tobacco slowly heated in a furnace at temperatures of 100–200 °C already generates an aerosol containing nicotine, clearly distinct from tobacco smoke [35], with water making a significant share of total particle matter (TPM), and CO, acetaldehyde, crotonaldehyde, formaldehyde, tobacco-specific nitrosamines NNK, and NNN detected at quantifiable levels (though much lower than in cigarette smoke). The aldehydes and CO were identified as byproducts of the pyrolytic decomposition of carbohydrates and tobacco biopolymers (cellulose, pectin, and sugars), while the nitrosamines were likely produced by evaporative transfer from the tobacco plant.

These thermophysical and thermochemical processes are essential for the design and operation of HTPs. However, to generate a more consistent aerosol and improve its delivery to users, HTPs heat suitably homogenized and reconstituted “tobacco sticks,” assembled in sheets and mixed with water, glycerol, and cellulose fibers. Four temperature ranges of slow heating of tobacco sticks were identified by the mass loss thermogravimetric curves [36,37]: a low-temperature region (30–120 °C) with moisture release leading to water evaporation; two intermediate temperature regions (120–250 °C and 250–370 °C) with thermal decomposition and evaporation, resulting in the emission of aldehydes, CO_2_, nicotine, water, and increasing CO as temperature increases; and a high-temperature region (370–550 °C) that continues further thermal degradation of residues in a nitrogen (oxygen-free) environment, but leads to the release of CO and nitrogen oxides in air, thus signaling the presence of oxidizing reactions characteristic of combustion processes. 

Two more recent studies by Cozzani et al. [38] and Eaton et al. [39] conducted TGA-FTIR analysis of the thermal decomposition of tobacco sticks, with the obtained emission profiles determined for each region of the thermogravimetric curves of the targeted HPHCs listed by regulatory agencies. A comparison between the experiments in an oxygen-free (nitrogen) environment and in an oxidizing environment (air) is crucial to prove the absence of combustion, which is produced by exothermic oxidative processes. For the entire range of temperatures below 350 °C (three of the four regions described above), the thermogravimetric curves of the nitrogen experiment are qualitatively similar to those in the presence of oxygen (air), which implies that no oxygen or oxidizing agents are required to sustain thermal decomposition, forming byproducts at these temperatures. At 350–400 °C (onset of the high-temperature region), the thermogravimetric curve of the experiment in nitrogen maintained the decomposition of residues and mass loss at a slow steady rate; however, the curve of the experiment in air showed a significant sudden drop in mass loss, indicating a sharp rate of oxygen consumption, together with a sharp increase in byproduct formation, especially some byproducts abundant in mainstream tobacco smoke (water, PAHs, phenols, and high levels of CO). The formation of NO_x_ at this stage occurred only in the experiment in air, which proves the emergence of oxidizing reactions. These are the physicochemical signals of the onset of combustion as the ignition temperature of tobacco (400 °C) is approached. These signals did not occur in the presence of air (or nitrogen) in the aerosols generated by heating tobacco at temperatures below 350 °C.

In addition to the absence of oxidative processes in an oxygen-free environment, the absence of combustion in aerosols from tobacco sticks can be appreciated from the difference between their particulate phase (TPM) and that of combustion-generated aerosols (such as tobacco smoke). Pratte et al. [40] examined the aerosol from IQOS heets and the smoke from the 3R4F cigarette with a thermodenuder (an instrument to separate and analyze the volatile content of aerosols up to 300 °C). Aerosols from the heets showed an 86% separation efficiency for glycerol, while 80% of the TPM of the smoke was neither evaporated nor separated. These outcomes clearly indicate a substantial difference between IQOS aerosols and 3RF4 smoke: the TPM of the former is composed of largely volatile droplets, and the TPM of the latter is mostly formed by solid particles or low volatile droplets with a mean diameter of 70 nm. These ultrafine, non-evaporating particles are characteristic of combustion-generated aerosols but are practically undetectable in IQOS aerosols. A similar characterization of TPM was found by Amoroz-Perez et al. [41] and Kärkelä et al. [42] using different experimental techniques, with aerosol and smoke from IQOS and 3R4C trapped in liquid media and analyzed by various techniques, including UV spectroscopy, GC-MS, and transmission electron microscopy. Pacitto et al. [43], in an independent study, also found IQOS aerosol particles to be predominantly volatile liquid droplets by estimating their volatile fraction from the change in the surface-to-volume ratio of (assumed) spherical droplets whose main diameters showed a mean 10-factor decrease when passing from 30 °C to 300 °C.

The temperature change during puffing is another phenomenon that illustrates the difference between HTP aerosols and tobacco smoke. Puffing involves forced convection air inflow, which in the temperature operation ranges below ignition in HTPs produces an endothermic cooling effect that can be measured as decreasing the temperature of the heater that is in contact with the tobacco stick. This is evidence that no exothermic oxidizing processes characterizing combustion occur (see the temperature graphs in Cozzani et al. [38] and Eaton et al. [39]). In contrast, puffing a CC triggers the consumption of oxygen, which releases heat and sustains the exothermic combustion process, increasing the temperature by hundreds of degrees to reach over 900 °C at the tip of the cigarette.

### 3.3. HTP Aerosols

The presence of glycerol in the reconstituted tobacco that forms sticks is essential for generating an aerosol that users should find a sensorially satisfactory replacement for tobacco smoke. As shown in experimental modeling of thermophysical processes, glycerol provides an efficient homogeneous nucleation agent for the formation of abundant aerosols [44]. In addition, when transitioning from laboratory experiments to the design of actual aerosol-generating devices, it is essential that reconstituted tobacco sticks are heated through an efficient equilibrium between thermal energy transfer (radiative or conductive) and convective airflow (inhalation).

While the IQOS and glo devices have different designs, both products share similar features (see specific details in Schaller et al. [45] and Eaton et al. [39]). Upon inserting the stick in the holder and turning on the battery, heat is supplied to the holder through a blade inserted into the tobacco plug, reaching between 200 °C and 350 °C (optimally 300 °C for the IQOS and 240 °C for the glo). These temperatures are sufficient to vaporize the volatile stick compounds below tobacco ignition. In both devices, the heating temperature is electronically controlled, automatically turning off the battery if the blade reaches 350 °C. 

Schaller et al. [45] conducted a comprehensive targeted analysis based on 54 priority toxicants identified by PMI, which include toxicants listed by the WHO, Health Canada, and the US FDA. They considered seven different puffing regimes and three climate conditions. In addition to water, glycerol, nicotine, total particulate matter (TPM), and nicotine-free dry particulate matter (NFDPM), 15 chemical families were targeted: volatiles, semi-volatiles, carbonyls, aromatic amines, nitrogen oxides, hydrogen cyanide, ammonia, epoxides, vinyl chloride, tobacco-specific nitrosamines, phenols, polycyclic aromatic hydrocarbons, nitrobenzene, metals (including mercury), and menthol. Their data reveal that for all puffing regimes and climatic conditions, all compounds found at levels significantly reduced (typically around 90% reduction) relative to their abundance in the mainstream smoke of the 3R4F reference cigarette. Similar results were obtained for the glo by Eaton et al. [39].

The non-targeted analysis by Bentley et al. [46] complements the findings of Schaller et al. [45]. Besides water, nicotine, and glycerol (which were quantified separately), the non-targeted screening found 529 compounds in concentrations ≥ 100 ng, constituting close to 100% of the total aerosol mass (96% confirmed with reference standards and only 0.2% assumed not detected). The most abundant compound of TPM is water, which, together with glycerol and nicotine, makes up about 95% of the TPM (in contrast, most TPM of tobacco smoke from the 3R4F cigarette is made of non-volatile liquid and solid particles). The total mass partition particulate/gas phase was 80%/20%, with 14 compounds making 80% of the gas phase mass. Of the 529 compounds, 68.6%/24.0% were exclusively in the particulate/gas phases, with the rest partitioned between the phases. These compounds were all found in the smoke of the 3R4F reference cigarette, the vast majority at significantly lower levels, but a minority was found at higher levels than in smoke. The latter include (for the non-menthol sticks) three compounds unique to the IQOS aerosol. However, the FDA evaluation of the PMTA application of the IQOS agreed with the claim of PMI that none of these compounds represents a toxicological concern (more discussion on this in Section 6.3).

### 3.4. HTPs vs. ECs

ECs also generate an aerosol that cannot be categorized as smoke, but the aerosol is generated by heating a liquid solution (the e-liquid) made of propylene glycol, glycerol, nicotine, flavorings, and water at temperatures between 180 and 270 °C, typically lower than those used in HTPs. Emission studies (when conducted appropriately, see further ahead) show that EC aerosols contain fewer toxic byproducts and have lower concentrations than HTP aerosols. This is expected not only because of the higher temperatures used in HTPs but also because the aerosol of HTPs is generated from tobacco sticks, which are a reconstituted form of biomass whose chemical composition is more complex than that of e-liquids; thus, it involves a wider variety of thermophysical and thermochemical processes. The main HPHCs found as byproducts in EC emissions typically reduce to 3–4 main aldehydes (formaldehyde, acetaldehyde, acrolein) that form from thermal decomposition of the solvents (PG and glycerol) and flavorings, with other HPHCs such as PAHs, TSNA’s, most VOCs (including CO), and metals not detected or barely above detection limits. However, while all of these HPHCs appear in HTP emissions at higher levels, they are still well below their abundance in tobacco smoke. 

Also, the high degree of standardization of the two main HTP products (IQOS and glo) greatly simplifies the procedures for appropriate aerosol generation by means of agreed puffing protocol standards. However, the appropriate laboratory testing of ECs (considering all combinations of devices, power settings, coil resistances, e-liquids, nicotine levels, and flavorings) is a more complicated endeavor. There is no recognized universal testing standard for EC emissions. Available standards (such as the CORESTA Method 81) are not appropriate for testing high-power sub-ohm devices. Hence, it is not surprising to find a wide diversity of results from EC emission studies in the literature, which points to the need to verify the compliance of laboratory studies with more stringent criteria of experimental quality, namely experimental reproducibility, testing devices in their proper operation ranges, and/or puffing parameters that are as close as possible to consumer usage. In two extensive reviews of 48 studies on EC emissions (12 focusing on metals [47], 36 on organic by-products [48]), we found that studies reporting concerning levels of toxic byproducts often failed to comply in various degrees with these quality criteria, which renders them totally or partially unreliable, while toxic byproducts remained negligible (well below their levels in tobacco smoke and toxicological markers) in all studies complying with these criteria.

## 4. Carbonyls in Emissions from HTPs (Independent Studies)

In the following section, we review eight studies by independent authors, focusing on their analytical methods and experimental quality. Of the eight independent studies reviewed, all examined HTP emissions and compared them with smoke analyses of commercial cigarettes and reference conventional cigarettes. Only one study by Uchiyama et al. [49] compared the emissions of the most popular commercially available HTPs (IQOS, glo, and Ploom) with the content of aldehydes and other toxic compounds. Six studies used the ISO 3308:2012 puffing regime (2 s puff, 60 s inter-puff, 35 mL puff volume) [50], whereas all other studies followed the ISO 20778:2018 or Health Canada Intense (HCI) regime (2-s puff, 60-s inter-puff, 55 mL puff volume) [51]. Six studies followed the DNPH derivatization mechanism. In general, as can be seen from Table 1, there is no homogeneity among the studies, either on the puff regimes used or on the analytical methods. The DNPH derivatization method was followed in almost all studies, with samples then analyzed using disparate methods. Although, in some cases, the following methods lack a proper description or do not follow standardized procedures, we can appreciate that the results follow a specific line, namely, an observed reduction in carbonyl compounds in the HTPs compared to the reference cigarettes.

### 4.1. Previous Reviews

Simonavicius et al. (2019) [25] reviewed 31 articles on Heated Tobacco Products published between 2009 and 2017. The purpose was to analyze the substantial differences between independent studies and those funded by tobacco companies (of the 31 studies they analyzed, 20 were funded by the tobacco industry) and to assess independent and tobacco industry-dependent research on toxic compound exposure during primary- and secondhand use. Overall, the authors found discrepancies in smoking regimens, reference products, and how the data were reported. In particular, regarding harmful and potentially harmful compounds (HPHC), the authors showed that some studies reported discrepant results compared with reference cigarettes. Notably, a comparison between studies on machine-generated emissions showed a greater reduction in exposure to HPHCs than that observed in randomized controlled trials, probably because machine-generated aerosols do not reliably reproduce human use.

Moreover, the large variety of methods used to measure and produce HTP-derived aerosols has facilitated the comparison of results from independent and manufacturer-funded studies. In cases where comparisons were possible, the sample sizes were too small to be considered statistically relevant. Given their observations, the authors highlighted the need to understand whether HTP production and analysis methods are suitable for reliably estimating emissions. In addition, they emphasized the need for the development and use of standardized protocols to allow for an optimal comparison of study results. 

Both independent and dependent studies have shown a reduction in HTP emissions within the 90% range of HPHCs. The studies funded by the companies that produced them provided no reason to consider erroneous characteristics in the analyses. In fact, the production of aerosols was always adequate, the analytical methods used were standardized, and the results were consistent with those obtained by independent authors.

El-Kaassamani et al. (2021) [26] provided the most extensive available summary of the literature on independent and industry studies of IQOS up to 2021 (Table S2, Supplementary Material of El-Kaassamani et al. paper). The list comprised 341 publications satisfying their inclusion criteria: 86 with authors affiliated with or supported by the PMI (25%), 246 by independent researchers (72%), and 9 by competing manufacturers (3%). Between 2015 and 2018, more studies were published by PMI-associated authors than by independent authors, but this trend reversed after 2018. Research associated with PMI before 2018 mainly consisted of comprehensive studies focused on generating evidence on emissions and preclinical data to support the IQOS application of an MRTP from the FDA, while independent research has responded on these topics but also focused on perception, awareness, and use prevalence trends, often expressed in brief reports and opinion pieces.

The authors confirm an overall agreement between independent and PMI research on estimations of nicotine yield of about 2/3 of 3R4F cigarettes, at least with the HCI regime (ISO 20778:2018) [51]. By looking at the puffing parameters, the authors report that larger puff times and volumes bear an influence on nicotine yields but no significant difference in carbonyl yields. They also mention an overall agreement between industry and independent studies on a significant reduction of the main aldehyde byproducts. The outcomes of all cited emission studies on nicotine, formaldehyde, acetaldehyde, and acrolein yields are summarized in Table S3 in their study for different puffing protocols, showing no statistically significant differences between PMI and independent studies (at least for the HCI regime).

The authors recognize that industry and independent studies agree on similar reductions of TSNAs, VOCs, PAHs, and other HPHCs with respect to the 3R4F under the HCI regime [51]. PMI studies did not notice a significant difference in toxicity relative to 3R4F cigarettes for different puffing regimes. However, they highlight that even in PMI non-targeted analysis [46], some compounds reach higher levels than in the 3R4F, with some of these compounds not included in the FDA list of 93 HPHCs. They cite a study [52] by St Helen et al. that revised the MRTP application of IQOS, finding that PMI did not supply data on 53 of the FDA’s HPHC list, of which 50 are carcinogens, while some of the 56 remaining toxicants absent from the FDA list (thus with possibly limited toxicity) were found at levels higher than in the 3R4F. St Hellen et al. provide the list of these compounds, showing that some known carcinogens have appeared at levels significantly above the 3R4F. El-Kaassamani et al. also cite independent studies that found higher levels of toxicants in HTP emissions, especially when using the ISO 3308:2012 [50] regime and compared with the 1R5F and ultra-light cigarettes instead of the 3R4F. These are interesting findings that we address in detail in Section 6.2.

### 4.2. Emission Studies

In the study by Auer et al. (2017) [53], the authors compared the emissions of VOCs, PAHs, and nicotine by comparing IQOS aerosols and conventional cigarette (Lucky Strike Blue Lights) smoke. The authors did not generate aerosols following a standard protocol or commercial puffing machine but used unidentified instruments designed at their site. As for the puffing regime, they followed the ISO standard for puffing volume (ISO 3308:2018) [48], with two puffs per minute and a total of fourteen puffs. As for the analytical method, it is not clear from the article what method was followed. The authors classified carbonyls as VOCs (as shown in the table in the article in question), which were analyzed by gas chromatography coupled with a flame ionization detector (GC-FID); however, they refer to a previously published article by Varlet et al. (2016) [54], where carbonyls were identified by derivatization on DNPH cartridges and then analyzed by HPLC-UV. Therefore, the analytical method was neither coherent nor reproducible. Furthermore, the authors did not specify how the samples were stored prior to analysis, how long the aerosol (if that was the case) was in contact with the DNPH cartridge, or how a blank analysis was performed. Regarding the analysis of PAHs, the simple result correlation approach is not the correct way to analyze the results of comparative studies. In fact, the two studies used a completely different method of analysis, in which a multitude of different variables could influence both the analysis and results and the measurement error associated with the particular measurement instrument should be considered. The authors also detected compounds not found in any other study, such as acenaphthene, but this could not be confirmed because of the lack of information about the analysis methods. However, even with all these flaws, the results clearly showed a percentage reduction of carbonyls in HTP emissions compared to conventional cigarettes (78% reduction for acetaldehyde, 18% reduction for acrolein, and 26% reduction for formaldehyde).

In the study by Farsalinos et al. (2018) [55], the authors determined the presence of carbonyl compounds in the emissions of the HTP product (IQOS) and compared them with smoke from conventional cigarettes (Marlboro Red) and aerosol from an EC (Nautilius Mini). The products were adequately described and allowed for the reproducibility of the experiment. The Health Canada Intense (HCI) [51] smoking regimen (puff volume of 55 mL, puff duration of 2 s, puff interval of 3 s) was used for aerosol collection. In addition, the authors used two non-standardized methods with a high-puff regime (puff volume of 80 mL, puff duration of 3 s, inter-puff interval of 30 s, puff volume of 90 mL, puff duration of 3 s, and inter-puff interval of 25 s). A shorter puff interval was adopted to obtain the maximum number of puffs that could be performed with a stick to the HTP. The ECs were tested at power settings of 10 W and 14 W. The carbonyls were derivatized with DNPH by passing the aerosols through impingers containing an acidic solution of DNPH and acetonitrile. Specifically, the authors collected smoke from two cigarettes, aerosols from two sticks of HTP, and 50 puffs of EC to allow for greater sensitivity in the detection of carbonyls. Analytical studies were performed using the CORESTA No. 74 method [56]. Compared with tobacco cigarettes, carbonyl levels in HTP emissions were found to be about 85–95% lower; specifically, 5.0–6.4 μg/stick of formaldehyde, 144.1–176.7 μg/stick of acetaldehyde, and 10.4–10.8 μg/stick of acrolein were detected. In contrast, no emissions of propionaldehyde and crotonaldehyde were recorded for ECs. The authors specify that μg/stick refers to μg/12 puff. Overall, the study was adequately executed in terms of both the description of the analytical methods and the methods used.

The purpose of Mallock et al.’s (2018) [57] study was to analyze HTP (IQOS, THS 2.2) emissions and compare them to those of conventional cigarettes (1R4F). They used the HCI [51] regime to produce aerosols. In particular, the authors analyzed the levels of nicotine, aldehydes, and other VOCs, as well as the total particulate matter (TPM). The authors used an LM4E smoking machine and analyzed four heating devices and two types of sticks. The four devices were IQOS, but differing in their degree of wear, whereas the authors report that they use two different types of sticks but do not declare which type. The aerosols produced were directly collected in impingers containing a DNPH solution and allowed to react for 30 min. The samples were analyzed using HPLC-DAD, performing chromatographic separation with a mobile phase of water and acetonitrile in a gradient, and the acquisition was performed at a wavelength of 360 nm. Confirmation of the presence of analytes was then performed using LC-MS/MS. The results of this study show that, for carbonyl compounds, HTPs exhibit a reduction in the range of 80% to 96% with respect to tobacco smoke. The results were reported as μg/stick, and the authors specified that they produced 12 puffs per stick.

The purpose of Uchiyama et al.’s (2018) [49] study was to analyze the gaseous and particulate compounds generated by HTPs. The results of the emission analyses were compared with those of the reference cigarettes (1R5F, 3R4F, CORESTA Monitor Cigarette (CM6)). In particular, what is extremely interesting in this paper is the comparison of the emissions of three different HTPs, IQOS, glo, and Ploom Tech, used with different compatible sticks for each device. The authors analyzed the emissions of unflavored sticks and sticks at two menthol concentrations. Aerosol production was achieved using an LX20 linear 20-port piston-type smoking machine, produced according to two regimes: ISO 3308:2012 [50] and HCI ISO 20778:2018 [51]. Aerosols were collected using a GF-CX572 sorbent cartridge and a fiberglass (GF) filter. This system, although not standardized, is intended to retain both the gas and particulate phases contained in HTP emissions and cigarette smoke. Specifically, carboxen 572 was used to retain the gas phase without impingers. Following elution, to study carbonyl compounds, a DNPH-enriched solution was added to the elution solution to perform derivatization and subsequent high-performance liquid chromatographic analysis. The authors performed a good characterization of the products used to produce the analyzed emissions; however, they used an unconventional method for trapping aerosols and, thus, a non-standardized method. Even the analysis of carbonyls, although well described, did not follow the CORESTA recommended method 74 [56]. The tests undertaken with the HCI regime produced for the three HTPs and reference cigarettes outcomes were comparable to other studies, but the test using the 1R5F cigarette as a comparator with the ISO regime produced interesting outcomes: several compounds were found in IQOS and glo emissions at levels higher than those of the 1R5F reference cigarette. We discuss these issues in Section 6.2.

Salman et al. (2019) [58] conducted analyses of carbonyls in aerosols of HTP (IQOS) devices and compared them with the emissions of conventional cigarettes (Marlboro Red). The custom blow puffing machine used to produce emissions was not described by the authors. The authors used two puffing regimes, HCI [51] (ISO 20778:2018) and ISO 3308:2012 [50], for comparison (six puffs). However, only carbonyl data from the HCI regime were reported. Derivatized carbonyls were extracted from the DNPH cartridges in a 90/10 (*v*/*v*) ethanol/acetonitrile solution and quantified using HPLC-UV. Separation was performed using a C18 column and water/acetonitrile/THF (6:3:1 *v*/*v*/*v*), water/acetonitrile (2:3 *v*/*v*), and acetonitrile as the mobile phase. Overall, the HTP samples contained lower carbonyl compound levels (472 ± 19 μg/session), 85% and 77% lower than those of the cigarette smoke samples. In the Discussion section, the comparison with the emissions of conventional cigarettes resulted in a reduction of more than 70% in the formation of toxicants. The authors argue that the quantified IQOS emissions contain more toxicants than urban air pollution, but this comparison is completely mistaken, as the authors failed to consider that IQOS emissions are intermittent and localized indoors, while urban air pollution is time-persistent and evaluated in large outdoor spaces.

The purpose of the study by Heide et al. (2020) [59] was to use single-photon ionization mass spectrometry (SPI-TOFMS) coupled with a linear smoking machine for the analysis of ENDS and conventional cigarette aerosols. Specifically, aerosols from an EC (PowerCig, Duvance, and Vype), an HTP (IQOS) with an electronic heating source, and an HTP (Eclipse) with a glowing piece of charcoal as a heating source were analyzed and compared with a reference 2R4F cigarette. The smoking machine followed a slight variation in the smoking method described in ISO 3308:2012 [50], with a 3 s puff, 30 s inter-puff, and 50 mL puff volume. Reference cigarettes were also studied under the HCI (ISO 20778:2018) [51] smoking regime. The smoking machine was directly connected to the SPI-TOFMS instrument through a heated metal capillary. This type of spectrometer generates ions following the absorption of ultraviolet photons and is analyzed by their time-of-flight through a free-field drift tube. In addition, a random smoking pattern with varying puff durations, volumes, and intervals between puffs was applied to compare different ECs. The results showed a decrease in the carbonyl content of EC aerosols compared with that of conventional reference cigarettes. Because it is a new and non-standardized method, the authors should have performed a blank analysis under the same analytical conditions to show whether the connecting tube used or other variables might have altered the results of the analysis during transport from the smoking machine to the spectrometer.

The study by Wang et al. (2020) [60] analyzed carbonyl levels in the emissions of HTPs by comparing them with those of “ultralight” cigarettes and 3R4F reference cigarettes. However, the authors did not describe the type of HTP and the brand/type of ultralight cigarettes, making the study impossible to reproduce (a serious flaw). However, for both the production of emissions and carbonyls analysis, they followed standardized methods. Aerosol emissions were measured using an automated smoking machine, according to the ISO 3308:2012 smoking regime [50]. Carbonyl analysis was performed using the CORESTA method No. 74 [56]. The overall results indicated that the levels of carbonyls emitted by HTPs were lower than those of the conventional reference 3R4F cigarettes, although they were higher than those of light cigarettes. Although the results are reported as micrograms per stick, it is not clear how many puffs correspond to one stick and how much aerosol volume was analyzed. However, according to Table 2 in their study, the unidentified “ultra-light” cigarette has a very low nicotine yield (0.12 mg/cigarette), much lower than the 3R4F and the unidentified HTP (0.707 mg and 0.55 mg per cigarette, respectively). As we show in Section 6.2, this very low nicotine yield makes the outcomes of this study unreliable and unrealistic since smokers smoking such cigarettes compensate by puffing much more intensely than machine puffing under the ISO 3308:2012 smoking regime.

In the study by Dusautoir et al. (2021) [61], the chemical compositions of emissions from an HTP, an EC, and a 3R4F reference cigarette were compared, and emissions were then used to perform cell exposure studies of human bronchial epithelial BEAS-2B cell line. The EC was a Lounge model with a 2.8 W Ni-Cr top-coil and 4.6 W power supply. The Mod box TC model, with 0.5 W kanthal bottom-coil and power supplies configurable from 7 to 50 W. Aerosols from the HTP (IQOS), EC, and smoke from 3R4F cigarettes were generated using a Vitrocell VC1 smoking machine with an HCI puffing regime [51]. Carbonyls were collected in two silica cartridges coated with DNPH. The DNPH cartridges were washed with acetonitrile and injected into the UHPLC-UV system. The separation was performed using water and acetonitrile as mobile phase and a C18 column. The acquisition was performed at a wavelength of 360 nm. Overall, the levels of carbonyls were lower in HTPs than those in conventional cigarettes. Regarding EC analyses, lower levels of carbonyls have been reported for the Lounge device (used at a power of 4 W), whereas high levels have been reported for the Mod box device (used at 30 W). These high carbonyl levels reflect the inadequacy of the HCI regime (conceived to test CCs) for testing sub-ohm EC devices at high powers (see [47,48]).

**Table 1:** Summary of analytical methods used in reviewed papers about carbonyls in emissions from Heated Tobacco Products.

Authors	Devices	Smoking Regimes	Analytical Methods	Derivatization Methods	Results Reported as
Auer et al. [53]	IQOS Lucky Strike Blue Lights	ISO 3308:2012	Incomprehensible	Incomprehensible	μg/cig
Farsalinos et al. [55]	IQOS Nautilius Mini Marlboro Red	ISO 20778:2018	CORESTA	DNPH solution	μg/stick: μg/12 puffs
Mallock et al. [57]	IQOS 1R4F	ISO 20778:2018	HPLC-UV LC-MS/MS	DNPH solution	μg/stick: μg/12 puffs
Uchiyama et al. [49]	IQOS glo Ploom Tech 1R5F 3R4F CM6	ISO 20778:2018 ISO 3308:2012	HPLC-UV	DNPH solution	μg/stick
Salman et al. [58]	IQOS Marlboro Red	ISO 20778:2018	HPLC-UV	DNPH-cartridges	μg/session
Heide et al. [59]	IQOS Eclipse PowerCig, Duvance Vype 2R4F	ISO 20778:2018 ISO 3308:2012	SPI-TOFMS	No derivatization	μg/puff
Wang et al. [60]	Unidentified HTP and ultra-light cig, 3R4F	ISO 3308:2012	CORESTA	DNPH solution	μg/stick
Dusautoir et al. [61]	IQOS EC Lounge NHOSS Mod Box 3R4F	ISO 20778:2018	HPLC-UV	DNPH-cartridges	μg/puff

**Table 2:** Summary of method validation used in reviewed in independent studies about carbonyls in emissions from Heated Tobacco Products. √ represents presence of analysis blanks. In contrast, x represents flawed or lack of blank samples.

Authors	Method Validation	Blank Analysis
Auer et al. [53]	Not detected	x
Farsalinos et al. [55]	LOD: 0.254 μg/collection for formaldehyde, 0.290 μg/collection for acetaldehyde, 0.395 μg/collection for acrolein, 0.440 μg/ collection for propionaldehyde 0.403 μg/collection for crotonaldehyde.	x
Mallock et al. [57]	The precision of the analytical method was calculated and reported as the mean value ± SD.	x
Uchiyama et al. [49]	LOD was calculated on the basis of the signal-to-noise ratios of 3: 0.76−17 μg/L. LOQ was calculated using a signal-to-noise ratio of 10: 2.5−58 μg/mL. Linearity: coefficients of determination greater than 0.9966. Reproducibility: expressed as the relative standard deviation (RSD), ranging from 1.9% to 5.1% (carbonyls) and from 0.23% to 4.4% (VOCs).	√
Salman et al. [58]	The precision of the analytical method was calculated and reported as the mean value ± SD.	x
Heide et al. [59]	The precision of the analytical method was calculated and reported as the mean value ± SD.	x
Wang et al. [60]	The precision of the analytical method was calculated and reported as the mean value ± SD.	x
Dusautoir et al. [61]	LOQ ranging from 6 to 15 ng/mL.	√

## 5. Studies on Heated Tobacco Products Funded by the Tobacco Industry

In this section, we review studies published by industry authors (PMI and BAT) between 2016 and 2018 on mainstream emissions from HTPs. These studies were cited in the review by Simonavicius et al. (2018) [25], while others were detected by screening the PubMed database. The studies revised below show great accuracy in the description of the analytical methods and derivatization mechanisms. Moreover, they collected a volume of aerosol produced using the same method (HCI ISO 207708:2018) [51]. Our review shows that despite the different analytical methods and experimental techniques summarized in Table 2, these studies show basic agreement with independent emission studies on the reduction of toxicant content in HTP emissions with respect to tobacco smoke.

In the study by Schaller et al. (2016) [62], the chemical composition, cytotoxicity, and genotoxicity of HTP (THS 2.2 IQOS) emissions were compared with those of conventional cigarettes. Specifically, an IQOS device with four types of sticks (two versions of the regular stick and two versions of the menthol stick) and a 3R4F reference cigarette were used. HPT emissions were produced following the HCI ISO 20778:2018 [51] protocol using a linear smoking machine LM20X. Twelve puffs were produced per HTP stick. Furthermore, emissions were generated using alternative puffing regimes according to observed human puffing behavior. The aerosol of the IQOS was collected using three micro impingers containing a DNPH solution, while the smoke of 3R4F was collected using two impingers connected in series. The solutions were then analyzed by LC-MS/MS using a mobile phase and a gradient solution of water/acetonitrile/isopropanol/tetrahydrofuran and acetonitrile. Overall, the results are in line with the literature and show a reduction of approximately 90% in the presence of HPHC in the emissions of HTPs, not only under normal puffing conditions (ISO) but also under extreme and non-standardized puffing regimes. A reduction of approximately 90% was also observed when comparing the cytotoxicity determined by the neutral red uptake assay and mutagenic potency in the mouse lymphoma assay. IQOS aerosol was not found to be mutagenic in the Ames test. In general, the study is well described, and the analytical methods are adequate; however, in the description of the carbonyl derivatization method with DNPH, the duration of the reaction and whether it was eventually quenched with a base has not been reported, making the method difficult to replicate.

The aim of Schaller et al.’s (2016) [45] study was to analyze the emissions of an IQOS (THP2.2) device used with different types of tobacco and 43 tobacco blends to understand their effect on the reduction of toxic substances. Once sticks containing the blends to be analyzed were produced, they were sent to the laboratory Labstat. They were conditioned following ISO protocols. Aerosols were generated using the HCI ISO protocol 20778:2018 [51] and tested according to the Health Canada T-104 method for the selected analytes. For carbonyls, derivatization was performed with a solution of DNPH in acetonitrile, followed by quenching with a Trizma base. The samples were then analyzed using an HPLC-UV method. The composition of the mixture had no significant effect on the formation of HPHC in aerosols. However, blends containing high proportions of nitrogen-rich tobacco produced higher yields of acetamide, acrylamide, ammonia, and nitric oxide. Moreover, from the data collected in this study, it was found that many HPHCs were found in the HTP aerosols released during tobacco curing. This suggests that HPHC levels can be further reduced by carefully selecting the type of tobacco used to produce sticks.

Jaccard et al. (2017) [63] evaluated the chemical composition of HTP IQOS (THS2.2) emissions by comparing it with smoke from different brands of conventional cigarettes on the market (instead of using a reference cigarette). The authors do not state what they are, but only the year and place of purchase. This does not allow the reproducibility of the method because the exact nature of the products is not stated. In particular, they compared the amount of HPHC to assess any reduction in the HTP. Aerosols and cigarette smoke were produced using a linear smoking machine, and 12 puffs were collected using the HCI ISO20778:2018 [51] puffing regime. In addition, all analytes in the HTP emissions and cigarette smoke were evaluated using only standardized Health Canada analytical methods (DNPH solution derivatization and HPLC-UV analysis).

In the study by Poynton et al. (2017) [64], the authors analyzed the emissions of a hybrid tobacco product (iFuse) that heats a tobacco cap through a liquid. The authors compared the emissions of this hybrid device with a 3R4F reference cigarette and electronic cigarette. Aerosol emissions for iFuse and the EC Vype ePen I were generated following the CORESTA-recommended regime (55 mL puff volume, 3 s puff duration at intervals of 30 s), and the instrument voltage was set at 3.6 V. The HCI [51] regime was used for the 3R4F reference cigarette smoke. Analytical investigations were performed following Health Canada (T-104) and Labstat International analytical methods. Because the duration of the conventional cigarette and that of the hybrid product were obviously different, the results were averaged for each puff. The results of this study showed that emissions of this heated tobacco hybrid product reduced toxicants by approximately 90% compared with the reference 3R4F cigarette. In addition, the emissions of hybrid tobacco products were more similar to those of EC aerosols than to those of conventional cigarettes.

In the study by Buratto et al. (2018) [65], a new LC-MS/MS method was used to study eight carbonyl compounds in aerosols trapped in phosphate-buffered salt solutions (PBS). This method was used to study smoke from conventional reference cigarette 3R4F and aerosols from an IQOS HTP (THS 2.2). The puffing protocol used was the HCI (ISO 20778:2018) [51]; however, different aerosol trapping methods were used depending on the analyzed phase. For all smoke-type samples (WS), the aerosol was trapped in a vial filled with phosphate-buffered saline; for gas vapor phase samples (GVP), the particulate aerosol phase was trapped on a glass fiber pad, while the vapor was trapped in a vial filled with phosphate-buffered saline placed after the filter. For 3R4F cigarettes, the GVP and WS fractions of the smoke generated by 10 items were accumulated and collected in 36 mL of PBS, and the GVP and WS fractions of the aerosol generated by 15 items were accumulated and collected in 25 mL of PBS. Finally, the carbonyls in the aerosols were derivatized with 2,4-Dinitrophenylhydrazine (DNPH) and HClO4 for 30 min and then quenched with pyridine. The derivatization reaction was carried out properly to avoid the formation of polyderivative compounds, and the preparation of the solution and progress of the reaction were adequately described. Carbonyl analysis was performed using ultra-high-performance liquid chromatography coupled with mass spectrometry (HPLC-MS/MS). Because of the novelty of using PBS solutions as traps for carbonyl compounds, the authors did not follow the recommended CORESTA analytical method, given the obvious diversity of the matrices. However, they performed a control experiment using a previously developed method [66] and compared the results with the values measured by Labstat International ULC using the official Health Canada method T-104 [67]. The results showed a decrease in the levels of carbonyls between the HTP and the reference cigarette 3R4F. The results were reported in μg/cig. However, although the results are expressed as micrograms per cigarette, the number of puffs relative to each cigarette was not specified, and only the volume of aerosol was generated.

The purpose of the study by Crooks et al. (2018) [68] was to analyze the aerosols emitted by HTPs (glo) to evaluate whether the addition of flavoring to sticks increases the risk of toxic substance production. In addition to chemical analysis, the authors also performed in vitro toxicology studies. Finally, they compared the results with those of 3R4F reference cigarettes. The choice of the aromas to be studied involved the use of those aromas for which no stability data had been found or that had been shown to be thermally unstable. The analytical studies were performed as described by Forster et al. (2018) [69] (see next paragraph, which is applicable to this study).

Forster et al. (2018) [69] assessed the levels of toxicants in HTP emissions using a conventional 3R4F cigarette as a comparison. The authors analyzed the emissions produced by the THP1.0 (glo) device with two different types of sticks (one mentholated and one non-mentholated) and compared them with the smoke produced by a reference 3R4F cigarette. Specifically, the emissions were produced following a smoking regime with the following characteristics: 55 mL volume, 2 s puff duration, and 30 s interval between puffs (HCI ISO 27709:2018) [51]. The authors performed stick conditioning according to the ISO standards. Eight puffs were collected for each stick. Reference cigarettes were conditioned and smoked according to ISO standards and smoked following the HCI regimen. Aerosols were produced by using a linear smoke machine. The analytical methods used by the Labstat International Laboratory were based on the Health Canada method for conventional cigarette smoke analysis. The authors stated that although the methods were not accredited for THP emissions, the laboratory performed validation, but the analytical laboratory performed additional validation to adapt and ensure compatibility with the THP aerosol matrix. The laboratory used 22 analytical methods for analyte quantification. Although some of these methods are Health Canada-certified, others were “in-house”. In particular, derivatization with PFBHA and GC-MS SIM was used to analyze the carbonyls. Correctly and optimally, the authors performed a blank analysis of the THP emissions and 3R4F smoke by vacuum blowing on the same linear machines during aerosol smoke collection. However, while methods performed using the Health Canada protocol are available, internal methods are not available (not even in the supplementary materials). This does not allow the reader to have all the analysis conditions used and does not make the analysis reproducible.

The purpose of the study by Eaton et al. (2018) [36] was to analyze the emissions of a heated tobacco product, glo (THP1.0), and compare them with those of a reference cigarette, 3R4F. Before analysis, the two products were conditioned according to the ISO protocols. Aerosols from the HTP and reference conventional cigarette smoke were produced following the HCI protocol [51]. The authors observed that for carbonyl compounds, there was a difference between the levels of acetaldehyde and formaldehyde and those of acrolein. The first two compounds showed a substantial reduction in HTPs compared to 3R4F cigarettes. This is in line with the results and expectations because the presence of these aldehydes is attributed to the sugars present in tobacco, which also undergo degradation in HTPs. Instead, acrolein and other volatile compounds produced during tobacco pyrolysis were present in the HTP aerosols at concentrations just above the detection limit.

The goal of the study by Bentley et al. (2020) [46] was to perform a non-targeted analysis of aerosols emitted by an IQOS (THS 2.2) HTP and compare the results with those of 3R4F reference cigarettes. Thus, the authors performed a broad analysis and detection of a large number of chemical compounds present in the HTP emissions and in conventional cigarette smoke. Non-targeted analytical methods have the advantage of providing the maximum coverage of all compounds in the matrix and complete and unbiased analysis. However, data interpretation can be extremely difficult because of the high number of false positives and negatives, as well as the possibility of detecting many unknown compounds. Another aspect to consider is the extreme difficulty of following the protocol. In fact, to maximize the detectability of all substances present, semi-quantitative analyses of gas chromatography coupled with time-of-flight mass spectrometry and liquid chromatography coupled with mass spectrometry with high resolution were performed on both the gas vapor phase and particulate matter. The method developed by the authors was as inclusive as possible. They generated aerosols of the HTP and smoke from 3R4F with a linear smoking machine using the HCI protocol [51]. For the separation of the two phases, they used Cambridge glass fiber, as recommended by several methods. The gas vapor phase (GVP) was then passed through impingers containing solvents that were not described by the authors. Gas vapor and particulate phase samples were analyzed following previously published protocols developed by the same authors. In general, some compounds known to be present in HTP aerosols and cigarette smoke were either not detected or detected in small quantities. This depends on the method used, which avoids the use of derivative solutions, and in any case, depends on the sensitivity and characteristics of the tools used for the analysis. In fact, a low-molecular-weight carbonyl compound such as formaldehyde, which is known to be present in cigarette emissions and ENDS, was not detected in this study, as well as compounds such as metals or high-molecular-weight molecules (Table 3 and Table 4).

**Table 3:** Summary of analytical methods used in reviewed papers about carbonyls in emissions from Heated Tobacco Products funded by tobacco industries.

Authors	Devices	Smoking Regimes	Analytical Methods	Derivatization Methods	Results Reported as
Schaller et al. [62]	IQOS 3R4F	ISO 20778:2018	LC-MS/MS	DNPH solution	μg/stick
Schaller et al. [45]	IQOS 3R4F	ISO 20778:2018	HPLC-UV	DNPH solution	μg/stick
Jaccard et al. [63]	IQOS CC 3R4F	ISO 20778:2018	HPLC-UV	DNPH solution	μg/stick
Poynton et al. [64]	iFuse Vype ePen I 3R4F	ISO 20778:2018	HPLC-UV	DNPH solution	μg/100 puff μg/stick
Buratto et al. [65]	IQOS 3R4F	ISO 20778:2018	LC-MS/MS	DNPH solution	μg/cig
Crooks et al. [68]	glo 3R4F	ISO 20778:2018	GC-MS SIM	PFBHA solutions	μg/stick
Eaton et al. [36]	THP 1.0 3R4F	ISO 20778:2018	HPLC-UV	DNPH solution	μg/stick
Forster et al. [69]	glo IQOS 3R4F	ISO 20778:2018	GC-MS SIM	PFBHA solutions	μg/stick
Bentley et al. [46]	IQOS 3R4F	ISO 20778:2018	Untargeted methods	No derivatization	μg/stick

**Table 4:** Summary of method validation used in reviewed studies funded by tobacco industries about carbonyls in emissions from Heated Tobacco Products. √ represents presence of analysis blanks. In contrast, x represents flawed or lack of blank samples.

Authors	Method Validation	Blank Analysis
Schaller et al. [62]	The precision of the analytical method was calculated and reported as the mean value ± SD. LOQ was calculated.	x
Schaller et al. [45]	The precision of the analytical method was calculated and reported as the mean value ± SD. LOQ was calculated.	x
Jaccard et al. [63]	The precision of the analytical method was calculated and reported as the mean value ± SD. LOD and LOQ were calculated.	x
Poynton et al. [64]	The precision of the analytical method was calculated and reported as the mean value ± SD. LOD and LOQ were calculated.	x
Buratto et al. [65]	The analytical method was validated according to the International Conference on Harmonization (ICH) guidelines.	√
Crooks et al. [68]	The precision of the analytical method was calculated and reported as the mean value ± SD.	√
Eaton et al. [36]	The precision of the analytical method was calculated and reported as the mean value ± SD.	x
Forster et al. [69]	The precision of the analytical method was calculated and reported as the mean value ± SD.	√
Bentley et al. [46]	The precision of the analytical method was calculated and reported as the mean value ± SD.	x

## 6. Discussion

### 6.1. Analytical Considerations

The purpose of this review was to describe the analytical methods used to characterize HTP emissions, focusing on studies that analyzed carbonyl compounds. These low-molecular-weight compounds are among the most toxic compounds in cigarette smoke and are generated by the degradation of carbohydrates naturally present in tobacco or in the solvent components of e-liquids. Seventeen studies that presented quantitative analyses of the carbonyls in Heated Tobacco Products were reviewed. We were careful to analyze both the independent studies and the studies funded by tobacco companies to cover the analytical territory as far as possible.

#### 6.1.1. Derivatization Methods

Several methods for derivatization of carbonyl compounds are described in this review. In general, the vast majority of studies (12 of the 17 reviewed) used the derivatization reaction with 2,4-DNPH for the formation of hydrazones, that is, adducts between carbonyl compounds and DNPH. The reaction of a carbonyl compound with DNPH is an addition-elimination reaction catalyzed by an acidic environment aimed at the formation of 2,4-dinitrophenyl hydrazones [70,71]. pH management is crucial for the quantification of carbonyl compounds in cigarette smoke. Too acidic pH promotes the condensation of carbonyl compounds, making accurate measurements difficult. Therefore, it is important to maintain the pH of the reaction solution at an appropriate level. In addition, the optimal reaction time for the derivatization of carbonyl compounds with DNPH is generally 30 min. This time allowed for the complete derivatization of all carbonyl compounds in the solution, preventing the formation of unwanted polyderivatized compounds. To stop the derivatization reaction, a solution of pyridine or Trizma base is usually added. This served to basify the solution, increase the pH, and prevent polyderivatization reactions. This step is critical for ensuring the stability of carbonyl derivatives and obtaining accurate quantification results. Specifically, 10 studies [36,45,49,55,57,60,62,63,64,65] used derivatization with DNPH solution, and it was generally common practice not to adequately describe aspects of this process, sometimes omitting the duration or quenching of the reaction (if it was performed and how).

Two studies used DNPH cartridges [58,61]. DNPH cartridges have some limitations compared with impingers containing an acidic solution of DNPH in acetonitrile [72]. These limitations include the following:

- Saturation: During analysis, the DNPH cartridges can become saturated with carbonyl compounds. This can occur during the same analysis or depends on the volume of aerosols passing through the cartridge. When a cartridge reaches saturation, it may not retain additional carbonyl compounds, thereby compromising the accuracy of the analysis.
- Condensate deposits: Condensates, such as water droplets or other substances in aerosols, may be deposited on the surface of DNPH cartridges. These deposits can hinder the ability of cartridges to efficiently retain carbonyl compounds, thereby affecting the amount of compounds detected.
- Secondary reactions with oxidants, such as ozone, can react with DNPH in cartridges, causing unwanted secondary reactions and interfering with the analysis of carbonyl compounds. These reactions can lead to biased results or overestimation of the presence of carbonyl compounds.
- Polymerization byproduct formation: In unsaturated carbonyl compounds such as acrolein, the possibility of polymerization byproduct formation during derivatization with DNPH has been observed. This polymerization may prevent the accurate identification and quantification of unsaturated carbonyl compounds, thereby introducing uncertainties in the analysis.

Despite these limitations, DNPH cartridges are still widely used as sampling and derivatization methods for the determination of carbonyl compounds in cigarette smoke and other matrices. The interpretation of the results must consider these possible sources of error and the specific limitations associated with the method used.

Two studies conducted by tobacco companies performed derivatization with O-(2,3,4,5,6-Pentafluorobenzyl)hydroxylamine hydrochloride (PFBHA) [68,69]. The reaction occurs through the nucleophilic addition of PFBHA to the carbonyl, forming an intermediate that subsequently undergoes a water elimination reaction to form an oxime derivative. This process is fundamental for the derivatization of carbonyl compounds to facilitate accurate analysis and measurement. Samples containing derivatized carbonyls were analyzed using GS-MS SIM. The selected ion monitoring (SIM) mode is used specifically for the quantitative analysis of matrices in which analytes are present in trace amounts. This method makes it possible to record the ion current at selected masses that are characteristic of the compound of interest by eliminating background signal interference [73]. 

In one study [53], it was unclear which derivatization method was used or whether this was actually performed. The remaining two studies did not perform derivatization. Heide et al. [59] sampled directly from the vaping machine to an SPI-TOFMS analytical instrument. Bentley et al. [46] performed a non-targeted analysis. These types of analyses are intended to characterize the matrix and confirm its components. Several overlapping analytical methods were used to cover the widest possible range of chemical classes. To avoid procedural problems with sample preparation, derivatization processes were not performed, and analytes such as carbonyls were identified after direct injection of the aerosols into the analytical instrument. As the authors correctly pointed out, some compounds whose analytical recognition techniques were too complex or required the use of an additional one were not analyzed. In addition, one of the most relevant carbonyl compounds, formaldehyde, was not detected in the analysis, probably because of its reactive nature and lack of derivatization, which may have reacted with other compounds in the aerosols.

#### 6.1.2. Analytical Methods

High-performance liquid chromatography with ultraviolet detection (HPLC-UV) is widely used to quantify carbonyl compounds in cigarette smoke and has been employed in 10 of the revised studies [36,45,49,55,57,60,61,63,64]. This method uses chromatographic separation and detection with a UV spectrometer to obtain precise and specific measurements. Reversed-phase C18 columns are commonly used as the stationary phase in HPLC-UV for the quantification of carbonyl compounds. C18 columns retain a wide range of carbonyl compounds and offer good selectivity. These columns tend to retain fewer polar compounds than more polar ones, allowing efficient separation of the compounds of interest. The use of a C18 column with high surface coverage further increased the selectivity of the separation, improving the ability to distinguish carbonyl compounds from other components present in the cigarette smoke matrix. To detect carbonyl compounds, it is common to set a wavelength of 360 nm using a UV spectrometer. This choice of wavelength prevents the detection of extraneous peaks that might exhibit higher absorbance at shorter wavelengths, ensuring specific measurements of the carbonyl compounds of interest.

Although HPLC-UV analysis is a standardized method (ISO 21160:2018) [74], it was designed for the analysis of smoke from conventional cigarettes. Liquid chromatography coupled with mass spectrometry is a method with greater sensitivity for the determination of carbonyls in ENDS aerosols. CORESTA has recently recommended a new method for the determination of carbonyl compounds in tobacco products (CRM 86) [75]. This could allow for a more accurate quantification of carbon and an adequate collection of analytical data.

#### 6.1.3. Blank Analysis and Sample Storage

- The blank method, or blank analysis, is an important practice in quantitative analysis to check for external contamination and ensure that the data obtained are indeed attributable to the sample being analyzed. Blank analysis involves the analysis of a matrix devoid of the analytes of interest, which is processed and subjected to the same steps as those used for the analysis of real samples. This enables the identification and assessment of contaminants that can affect the analytical results.
- In the context of the reviewed studies, only five performed or described blank air analyses [49,61,65,68,69]. This means that only a small percentage of the studies considered possible external contamination and performed controls to exclude contamination from the analysis results. Blank analysis is particularly important when studying volatile compounds in air because the air itself can be subject to contamination from a variety of environmental sources. Verification that the data are derived only from the sample and not from external contamination is critical for ensuring the reliability of the analysis results. Blank analysis provides an additional check to identify and correct for any contamination that may compromise the accuracy and interpretation of the data obtained. In the remaining studies, it is unclear whether the blank analysis was performed at all or that its mention was omitted in the final article.
- In the context of the reviewed studies, there seems to be a lack of information regarding the storage conditions of the samples before analysis. This lack of detail may be a limitation in understanding and reproducing the results. In fact, only a few studies have specified that characteristic ISO conditioning was performed. The storage conditions for aerosol samples may vary depending on the compounds of interest and the analysis objectives. However, some general considerations include the following.
- Temperature storage: Aerosol samples should be stored at appropriate temperatures to preserve the analyte stability. Depending on the nature of the carbonyl compounds and solvents used, refrigerated or frozen temperatures may be necessary to prevent the decomposition or volatilization of the analytes.
- Protection from light: Some compounds may be sensitive to light and undergo undesirable photochemical reactions. Therefore, aerosol samples should be protected from direct light or stored in opaque containers to prevent alteration of analytes.
- Prevention of internal secondary reactions: It is important to ensure that collected aerosol samples are stored in a manner that minimizes the possibility of unwanted chemical reactions within the samples. This may require the use of inert containers or the use of appropriate chemical stabilizers.
- Storage time: It is important to consider the length of time that aerosol samples can be stored prior to analysis. Some compounds may be subject to degradation over time; therefore, it is necessary to assess the stability of the analytes of interest and define appropriate storage times to avoid alteration of the results.

In conclusion, the lack of information on the storage conditions of aerosol samples limits the understanding and reproducibility of the study results. For the proper interpretation and reproduction of data, it is essential to know and report the storage conditions of the samples, including factors such as temperature, light, and storage time. This ensured that the results obtained were representative of the initial conditions and were not affected by any unwanted reactions or deterioration of analytes during sample storage.

#### 6.1.4. Influence of Puffing Regimes

Despite the extreme difficulty of having a puffing regime that can reproduce the real use of HTP users, and more generally, ENDS, two standardized regimes, HCI and ISO, were conceived. The ISO 3308:2012 [50] smoke regime uses a smoking machine with standardized settings, which include a puff volume of 35 mL, a puff duration of 2 s, and an interval between puffs of 60 s. This regimen was developed to mimic the smoking modes of a wide range of human smokers. The HCI smoke regime is more intense than that of the ISO. A smoking machine with a puff volume of 55 mL, puff duration of 2 s, and interval between puffs of 30 s. In addition, in the HCI regime, cigarette filters were blocked with adhesive tape to prevent air from entering the filter vents [51]. This affects the filter ventilation and paper porosity, reducing the amount of tar, nicotine, and carbon monoxide (CO) measured using the ISO regime.

A study conducted by Pauwels et al. (2018) [76] shows the influence of the smoking protocol, particularly on the production of carbonyl compounds. It was highlighted that the highest puff volume in combination with a shorter puff interval in HCI leads to increased aldehyde yields in all cigarette brands when smoked according to HCI. A similar effect was observed when non-standardized smoking regimes were studied [77]; a higher volume resulted in higher detection of nicotine, tar, and CO.

### 6.2. Challenges to HTP Safety Consensus: More HPHCs Than in Tobacco Smoke?

The positioning statement of the World Health Organization (WHO) [78,79] (see Section 6.4 for more detail) mentions the presence of toxins in IQOS and HTP emissions found at levels higher than in CC smoke as a detrimental argument for their relative safety. This point is important since it represents a challenge to the consensus on HTPs exposing users to significantly lower levels of HPCPs in comparison with CC smoke. This questioning was also emphasized in the abstract and in the discussion of the literature in the review that we revised in Section 4.1 by El-Kaassamani et al. [26], who state in their abstract conclusion that “independent studies and examination of PMI’s data showed significant increases in other emissions from and beyond the Food and Drug Administration’s harmful and potentially harmful constituents list”. Therefore, it is important to examine these findings and discuss their implications.

The existence of compounds in IQOS aerosols at levels above tobacco smoke has never been denied by PMI. The first independent authors raising this issue were St Helen et al. [52] in 2018, commenting that “it appears that IQOS reduces exposure to some toxicants but elevates exposure to other substances”, referring to a list of compounds that PMI presented in an addendum to its MRTP application, containing 113 constituents of IQOS emissions that include 56 of the 58 constituents on the PMI-58 list, plus 57 not appearing in this list. Of the latter 57 compounds, 56 had levels above 3R4F (median 154% but up to 13,650% higher), 22 of them at least 200% higher, 7 at least 1000% higher.

This finding seems alarming, but it needs to be placed in its proper context: a compound having a much larger abundance in IQOS aerosol than in 3R4F smoke does not necessarily imply large toxicity, especially when these 56 constituents appear in minuscule quantities (in other words, 1000 or more times a negligible quantity is likely to be still negligible). In Table 1 of their study, St Helen et al. [52] provide the full list of the 113 compounds in the mentioned addendum, comparing their yields (μg/stick) with those found in the smoke of the 3R4F (μg/cigarette). Although, as claimed by St Helen et al., many in this list of 113 compounds are toxic or potentially toxic or even carcinogenic, it is important to infer or estimate their level of toxicological concern given the minute quantities they appear in this list. Since there is no information on the inhalation toxicity of most listed compounds, we show in Table 5 that the exposure doses for four compounds in the list (1,4-Dioxane, furanmethanol, glycidol, and furfural) for which toxicological data exist are a tiny fraction (around 1/1000) of the thresholds of occupational and environmental safety. 

Notice from Table 5 that there is no toxicological concern, even for the compound 1–4 Dioxane found in IQOS emissions at levels 13650% higher than in the smoke 3R4F cigarette. Even in this most extreme case, the daily inhalation dose is 1/3000, the strict threshold given by the MRL of the ATSDR-CDC. The technical documentation of the US FDA (see Section 6.3) reveals that during its evaluation of the IQOS, the agency was aware of the existence of HPHCs in its emissions at levels above those of the 3R4F smoke but did not consider this to be a serious challenge in its evaluation and granting of the modified exposure order (see further discussion in Section 6.3 of arguments expressed in pages 20–26 of [80]).

It is worth mentioning that describing HTP emission outcomes as percentages above the levels in the 3R4F cigarette can be misleading. For example, El-Kaassamani et al. [26] commented that in a study of Juul pods [81] the emission from IQOS measured glycidol (a carcinogen) at levels 400% above those of 3R4F. This is alarming, but the actual measured glycidol quantities show that there is no reason for concern. From Table 5 of [81], the glycidol levels are 0.00084 (3R4F) and 0.0043 (IQOS) normalized with nicotine yield (0.172 mg/puff), leading to 0.14 μg/puff (3R4F) and 0.73 μg/puff (IQOS)—considering 300 daily puffs (150 puffs in 8 h) leads to a glycidol dose from IQOS of 0.109 mg, which is well below (3 orders of magnitude) the occupational safety threshold of 40.67 mg shown in Table 5. 

The concern voiced by St Helen et al. in 2018 [52] was echoed in the extensive 2021 review summary of the literature on IQOS by El-Kaassamani et al. [26] (see Section 4.1). These authors cite more recent studies that have found HPHCs in HTP emissions above their levels in various CCs, especially when using the ISO 3308:2012 and/or comparing with the 1R5F cigarette or “ultra-light” cigarettes. However, using outcomes from machine puffing with the low-intensity ISO 3308:2012 regime might produce an unrealistic comparison since observational and demographic studies [76,77,82] report that this puffing regime underestimates toxicant exposure and is unrepresentative of realistic cigarette puffing (much less representative than the HCI regime).

Wang et al. [60] (revised in Section 4.2) examined emissions of an unidentified HTP in comparison with the 3R4F cigarette and unspecified “ultra-light” cigarettes, all tested with the ISO 3308:2012 regime. Four compounds (acetaldehyde, propionic aldehyde, butyral, and crotonaldehyde) were found in the HTP at levels above those of the “ultra-light” cigarettes. However, these findings are not reliable, not only because the products are unidentified, but because the “ultra-light” cigarettes had a very low nicotine yield: 0.12 mg per cigarette (vs. 0.707 mg/cigarette for the 3R4F and 0.55 mg for the unidentified HTP). There is a large amount of evidence [76,77,82,83,84,85,86] showing that when smoking low nicotine (and low tar) cigarettes, smokers puff more intensely (longer puff times, shorter inter puff times, and larger puff volumes), a puffing pattern that greatly differs from machine puffing this type of cigarettes with the low-intensity ISO 3308:2012 regime (besides this, the authors provide no information of the number of puffs used for these cigarettes). Therefore, this comparison is completely unrealistic and unrepresentative.

**Table 5:** Four HPHCs found in iQOS at levels above 3R4F. These compounds appear in Table 1 of [52], the list of 113 compounds supplied by PMI to the FDA in an addendum. Inhalation dose computed for average adult daily breathing 20 m^3^ of air. The TWA are time-weighed averages of a lifetime 8 h work journey, while MRL ATSDR is daily chronic exposure. Notice that there is no concern for the toxicity of these compounds in iQOS, as inhalation doses are about 1/1000 the safety threshold.

Compound	iQOS µg/Stick	3R4F µg/cig	% Excess in IQOS	Toxicological Marker mg/m^3^	Inhalation Dose iQOS	Safety Threshold
2-ethyl-5-methyl-1,4-Dioxane	0.055	0.0004	13650	MRL ATSDR-CDC 0.16 [87]	1.1 µg/day	3.19 mg/day
Furanmethanol	39.2	7.0	460	OSHA TWA 8 h 40 [88]	274 µg/8 h	266 mg/8 h
Glycidol	5.71	1.76	224	CalOSHA TWA 8 h 6.1 [87]	39.9 µg/8 h	40.67 mg/8 h
Furfural	31	25.9	20	ACGIH TLV © 8 h TWA 0.8 [87]	217.7 µg/8 h	5.33 mg/8 h

El-Kaassamani et al. report another study, Uchiyama et al. [49], that found HPHCs in HTP emissions above their levels in CC smoke, but only when testing them in the ISO 3308:2012 regime and with comparison with the reference cigarette 1R5F. We list these compounds and their yields in Table 6.

However, as mentioned before, the machine puffed ISO 3308:2012 regime under-estimates smokers’ toxicant exposure, and the 1R5F cigarette has a very low nicotine yield (0.120 ± 0.43 mg/cigarette, though still within experimental values [76,77]). Nevertheless, the exposure (TWA 8 h) of all compounds found in IQOS and glo above the smoke of 1R5F were below occupational toxicological limits, with the exception of diacetyl (although the toxicological marker used for this compound is also more stringent). 

In summary, the presence of some compounds in HTP emissions at higher levels than in the smoke of reference cigarettes does not (necessarily) translate into concerning levels of toxicity in these emissions. This depends on the concentrations and inhalation doses of these HPHCs. Detection of such HPHCs does not constitute a robust questioning of the relative safety of HTPs, as these compounds appear in minuscule quantities, and a risk assessment needs to consider the full chemical content of the emissions. Nevertheless, there are no inhalation toxicological data on many of these compounds, making it important to monitor the effects of HTP emissions through preclinical and clinical effects as the usage of HTPs as CC substitutes continues to expand.

### 6.3. The US FDA and Other Regulatory Agencies

The Food and Drug Administration (FDA) of the US conducted what perhaps can be the most exhaustive technical evaluation of an HTP product, the IQOS, manufactured by PMI, which applied in December 2016 for the status of Modified Risk Tobacco Product (MRTP) with both the “modified exposure” and “modified risk” orders under, respectively, sections 911(g)(1) and 911(g)(2) of the Federal Food, Drug, and Cosmetic Act (FD&C Act). As a result of this evaluation, the US FDA recognized the claim by PMI that IQOS “heats tobacco without burning it”, and thus it is “appropriate for the protection of public health”. The US FDA approved the marketing of IQOS, but only in the “modified exposure” order, denying the “modified risk” order. The document [80] elaborated by the evaluators of the submission by PMI, the Tobacco Product Scientific Advisory Committee (TPSAC), provides a full summary of technical issues and regulatory criteria behind their evaluation.

The technical and regulatory evaluation of IQOS by the US FDA is one of the most important endorsements of a THR product. Unfortunately, it is often misunderstood, as if only receiving the modified exposure order (sections 911(g)(2)) and not the modified risk order (sections 911(g)(1)) is a sort of shortcoming or permanent devaluated status. This is not the case; the modified risk and exposure orders are linked as part of the regulatory pathway of the US FDA for tobacco products, with the modified exposure order being a necessary (but not sufficient) condition for the modified risk order. In fact, for the US FDA to grant a modified risk order, PMI would have to provide evidence of long-term actual usage of the IQOS, which, according to the TPSAC, was not achieved by the evidence supplied by PMI [80]. This requirement implies (among other factors) epidemiological evidence that a novel product can hardly provide, but such evidence would be extremely unlikely to emerge without evidence that users switching completely from cigarettes to IQOS do achieve a significantly reduced exposure to HPHCs, evidence that (according to the TPSAC) PMI was able to provide, thus fulfilling the requirements for the modified exposure order that the IQOS received. 

The arguments stated above are explained in the technical justification of the evaluation outcome on pages 11 and 69–71 of [80]. First, the justification of the modified exposure order (page 11):

*With respect the exposure modification order request, the applicant has demonstrated that the products sold or distributed with the proposed modified risk information meet the standard under section 911(g)(2) of the FD&C Act, **including that a measurable and substantial reduction in morbidity or mortality among individual tobacco users is reasonably likely in subsequent studies**, and issuance of an order is expected to benefit the health of the population as a whole taking into account both users of tobacco products and persons who do not currently use tobacco products. [our emphasis]*

With this unequivocal statement, the US FDA expresses that an “exposure modification” given to IQOS comes with a reasonable likely reduction in morbidity and mortality. The justification for the denial of the modified risk order is explained in the following paragraph, not as a shortcoming or defect, but as the first necessary stage of a regulation pathway (pages 69–70):

*In short, unlike the section 911(g)(1) standard, which requires scientific evidence showing actual risk reduction (e.g., a finding that the product, as actually used by consumers, will significantly reduce harm and risk to individual users; a finding that the product, as actually used by consumers, will benefit the health of the population a as whole), section 911(g)(2) establishes a lower standard, which allows FDA to issue an order when risk reduction has not yet been demonstrated but is reasonably likely based on demonstrated reductions in exposure (e.g., a finding that a reduction in morbidity or mortality among individual users is reasonably likely in subsequent studies; a finding that issuance of an order is expected to benefit the health of the population as a whole).*

These paragraphs make abundantly clear that the US FDA would not have granted the modified exposure order if the evidence so far supplied would cast significant doubt on achieving, with time, the requirements of the modified risk order. 

Other regulatory agencies have also evaluated HTPs and the IQOS in particular. The German Federal Institute for Risk Assessment (BfR) commissioned a study by Mallock et al. [57] (SFP Grant no. 1322-53). The UK government commissioned a report by the Committee on Toxicity of Chemicals (COT) in Food, Consumer Products and the Environment [18], Public Health England [19], and the National Institute for Public Health and the Environment in The Netherlands [20]. Despite the different regulatory approaches, all these agencies have reported conclusions that converge with the US FDA evaluation. Notwithstanding uncertainties and insufficient research, the emissions of the devices expose users and bystanders to a significantly reduced content of HPHCs with respect to tobacco cigarette smoke. While their recommendations within the THR context are varied and not as detailed as those of the US FDA, all assume in nuanced form a benefit to smokers who adopt HTP usage in substitution of tobacco smoking.

### 6.4. The WHO

The World Health Organization commissioned a Study Group on Tobacco Product Regulation to undertake a technical evaluation of HTPs [92], resulting in an extensive technical review of the literature (industry and independent studies). In its conclusions (page 37), this document upholds the consensus that HTP emissions expose users to significantly fewer toxicants than conventional cigarettes. However, other non-technical communications by the WHO, such as the latest HTP Information Sheet [78] and the positioning statement of the WHO on the US FDA evaluation [79], express a much more precautionary and skeptical approach towards the evidence on reduced exposure to toxicants and on how HTPs should be regulated. Both WHO communications issue the following statements:

- The US FDA statement noted that “Even with this action [MRTP modified exposure order], these products are not safe nor “FDA approved”;
- The US FDA authorization rejected claims that the use of the product is less harmful than other tobacco products or reduces health risks;
- The WHO reiterates that reducing exposure to harmful chemicals in Heated Tobacco Products (HTPs) does not render them harmless, nor does it translate to reduced risk to human health;
- Some toxins are present at higher levels in HTP aerosols than in conventional cigarette smoke, and there are some additional toxins present in HTP aerosols that are not present in conventional cigarette smoke. The health implications of exposure to these are unknown.

These statements misunderstand the FDA evaluation (see detailed explanation in Section 6.3). We address the first three points in the bulleted list above, as we have dealt with in Section 6.2, with the potential toxicity of toxins found at levels above CC smoke (the last point in the bulleted list). By denying the modified risk order, the US FDA did recognize that evidence supplied for the modified exposure order does not prove that usage of IQOS reduces harm in users, something that would require long-term evidence to make it evident. However, (as explained in Section 6.3) the US FDA technical documentation [80] clearly states that the evidence supplied for the modified exposure order made it plausible and likely that such long-term evidence should emerge in future studies. While the WHO positioning statement correctly quotes the US FDA emphasizing that the MRTP-modified exposure order does not mean that IQOS is “safe” or “FDA approved”, these statements must be taken in their full context since the US FDA has also emphasized that “There are no safe tobacco products, and those who do not use tobacco products should not start”. Evidently, no evaluation or communication (not even from the industry) will ever consider a tobacco product as “safe”, not to mention being “harmless”. However, these absolute safety remarks do not deny the benefits from the relative safety with respect to cigarette smoke that justified the US FDA granting the modified exposure order, as can be appreciated in the following statement issued in 2018 [13]:

*“Data submitted by the company shows that marketing these particular products with the authorized information could help addicted adult smokers transition away from combusted cigarettes and reduce their exposure to harmful chemicals, but only if they completely switch”. (Mitch Zeller, J.D., former director of the FDA’s Center for Tobacco Products)*

This clearly places the appropriate harm reduction context of the US FDA evaluation, namely, the IQOS or any product with modified exposure order is not “safe”, so it is not recommended to the general population, only for smokers as a full substitution of tobacco cigarettes, the most harmful form of nicotine delivery. In fact, contrary to the WHO statements quoted above, the paragraphs we have quoted from the US FDA do recognize (at least potentially with high likelihood) that IQOS usage is safer than smoking.

### 6.5. HTP Aerosols Are Not “Smoke”

Two studies, Auer et al. [53] and Uguna and Snape [93], issue ambiguous claims that HTP aerosols can be categorized as a form of smoke, claims largely sustained by speculative arguments that ignore the abundant research on thermophysical and thermo-chemical processes in heating tobacco without combustion, which we summarized in Section 3.2, which objectively shows that aerosols generated by HTP operating in their normal conditions are not “smokes” under any accepted physicochemical definition of this term. 

Although Auer et al. [53] suggest that HTP aerosols are “smoke by another name”, their arguments are speculative and lack any support for their actual data (as we show in Section 4.2, this study exhibits serious methodological flaws). A recent paper by Uguna and Snape [93] cites Auer et al. and advances the claims that IQOS emissions can be “either an aerosol or a smoke”, but the authors do not support this claim by any experimental evidence, besides not citing or making any connection to the abundant literature that we summarized in Section 3.2. This paper is an attempt to update and expand the speculations of Auer et al. [53]. Their claim that IQOS emissions fit the concept of smoke is based on four arguments: detection of solid ultrafine particles in the TPM, detection of the same HPHCs found in tobacco smoke (such as CO), the existence of pyrolysis and other processes that also occur in the generation of mainstream CC emissions (see Section 3.1), and the possibility that some spots in tobacco sticks might be heated above 350 °C. These arguments contain a kernel of truth but do not prove the authors’ claims: while solid particles and CO might be present in IQOS emissions, its TPM is overwhelmingly made of volatile liquid droplets [40,41,42,43], and CO and other compounds are found at much lower levels than in tobacco smoke [45,46,57,61]. Pyrolysis and distillation occur in generating IQOS aerosol, but as endothermic processes are irrespective of the presence of oxygen, while in the formation of tobacco smoke, they are preceded by a highly energetic, oxidizing, and exothermic ignition (see Section 3.2 and Section 3.3). The possibility of heating tobacco sticks above 350 °C is pretty speculative without any empirical evidence. Finally, the authors claim that the relative abundance of HPHCs in IQOS emissions is underestimated by a factor of 3 because the mass of the sticks is 1/3 the mass of a cigarette. However, the important parameter in safety comparison with tobacco smoke is the relative concentration in the generated aerosol/smoke, not the mass of the tobacco material. Neither Auer et al. [53] nor Uguna and Snape [93] provide a valid argument to justify considering IQOS emissions as smoke (see the rigorous essay by PMI scientists on the lack of combustion in IQOS [30]).

### 6.6. Evaluation of the Studies

After the considerations extensively described in this discussion have been made, we report a table summarizing our assessment of the reliability of the reviewed studies. In particular, consideration was given to the information provided by the authors to enable experimental reproducibility, the analytical method and its adequacy, the method of aerosol production (with different puffing regimes), and the evaluation of blank samples. Table 7 shows the results of our assessment of the reliability of the reviewed studies, assigning a score to each of the following characteristics: 1 if the requirement was met, 0 if the requirement was not met, and ½ for studies that partially met the requirement. We used a “traffic light” coloring of the scores, with “Reliable” (green) for scores of 3.0 or higher, “Partially Reliable” (yellow) for scores between 2.0 and 3.0, and “Unreliable” (red) for scores below 2.0. Overall, only one study was found to be completely unreliable, while three studies were found to be partially reliable due to the lack of specification of the units of measurement used and/or poor validation of the methods. The rest of the studies were found to be reliable, with some minor flaws that did not substantially affect their outcomes.

## 7. Conclusions

This review provides an in-depth revision and technical evaluation of analytical methods and experimental procedures of 17 studies (industry and independent) focused on the detection of carbonyls, describing and evaluating different carbonyl analysis methods in HTP aerosols. Due to variations in HTP regulations and cultures between nations, the 17 research studies have different backgrounds and sets of analytical techniques. The main outcome of our review is the finding that, despite the diversity of analytical methods and experimental procedures, practically all studies sustain the general consensus that HTP emissions contain worrying HPCPs but at substantially lower concentrations than tobacco smoke. Our revision clearly supports the validity of the experimental evidence behind this consensus.

The PRISMA search of the literature and the methodological approach that we followed are depicted in Figure 1 and Figure 2. To provide a theoretical context, we also reviewed (in Section 3) the literature on thermophysical and thermochemical processes behind the process of heating tobacco without combusting it, which provides the main technical justification for the design and operation of HTPs. We also reviewed and summarized studies (industry and independent) that focused specifically on the constituents of HTP aerosols. In general, HTP emissions contain far fewer compounds than tobacco smoke and exhibit, on average, a 90% reduction in HPHCs. The TPM of HTPs is dominated by water and has a high volatile content, whereas the TPM of tobacco smoke is mostly composed of low-volatile droplets and solid particles. We also provide an extensive discussion (Section 6.2, Section 6.3 and Section 6.4) on challenges to the consensus of HTP relative safety with respect to CC and of the evaluations of HTP emissions (especially IQOS) by the US FDA, other regulatory agencies, and the WHO. In spite of the different approaches, precautionary concerns, and other nuances, all stake-holders converge on a broad consensus behind the significant reduction of HPHC content in HTP emissions relative to tobacco smoke.

Because HTPs are relatively standardized products, the aerosol generation techniques and analytical methods used to test their emissions tend to follow a relatively standardized pattern. Consequently, the quantification of the presence of carbonyls and other byproducts tended to converge to qualitatively similar outcomes. This is an important difference with studies testing EC emissions, which often leads to widely diverging outcomes for carbonyls and other toxic byproducts.

It is important to remark that the status of MRTP under the “reduced exposure” order, granted by the US FDA after an extensive evaluation of IQOS, represents an important endorsement of a THR product. However, we do recognize the existence of controversy and the need for further research and more data. We also need to monitor the effectiveness of HTPs within the THR approach to address the harmful effects of smoking and to assist in smoking cessation. Emission studies showing a reduction in exposure to HTHPs relative to tobacco cigarettes constitute the first step in assessing the health effects of HTPs in actual users, especially the long-term effects. This first step should be followed by studies on biomarkers and preclinical and clinical studies.

## Figures and Tables

**Table 6:** HPHCs in iQOS and glo emissions were found at levels above the 1R5F reference cigarette. Concentrations were obtained by Uchiyama et al. [46] in the ISO 3308:2012 regime. Notice that, with the exception of diacetyl, the exposure (TWA 8 h) to all these HPHCs is well below the occupational safety limit. “NA” stands for toxicological marker not available.

Compound	µg/Stick	µg/Cig (1R5F)	µg /Cig (3R4F)	Toxicological Marker mg/m^3^	Exposure Dose	Safety Threshold
**IQOS**						
Acetol	65, 81, 84	4.6 ± 0.78	22 ± 1.4	NA	0.43–0.56 mg/day	NA
Butanal	15, 15, 15	8.7 ± 0.82	26 ± 1.1	NA	0.1 mg/day	NA
Diacetyl	42, 49, 41	34 ± 0.9	120 ± 8.9	0.017 TWA lifetime 8 h (NIOSH) [89]	0.27–0.32 mg/8 h	0.11 mg/8 h
Glyoxal	3.6, 4.5, 4.8	2.2 ± 0.44	8.9±0.55	CAL/OSHA PEL 0.1 [90]	0.024–0.032 mg/8 h	0.66 mg/8 h
1-valeraldehyde	8.8, 8.4., 8.3	4.6 ± 0.96	22 ± 1.6	175 NIOSH REL [87]	0.055–0.058 mg/8 h	1166.67 mg/8 h
**glo**						
Acetol	40, 46, 49	4.6 ± 0.78	22 ± 1.4	NA	0.26–0.32 mg/8 h	NA
Furfural	68, 79, 150	1.2 ± 0.35	14 ± 2.0	ACGIH TLV © 8 h TWA 0.8 [87]	0.45–1.0 mg/8 h	5.33 mg/8 h
Methylglyoxal	1.4, 4.3, 4.3	3.0 ± 0.55	14 ± 1.1	NA	0.009–0.028 mg/8 h	NA
Pyridine	16, 14, 1.2	1.1 ± 0.4	9.5 ± 1.1	15 ACGIH TWA 8 h [91]	0.008–0.106 mg/8 h	100 mg/8 h

**Table 7:** Assessment of the reliability of the revised studies. The symbols √ and x represent one and zero score points, respectively, whereas 1/2 represents half a point. The points were given based on the fulfillment of the condition. Reliability is indicated through a color-coded system where “Reliable” (green) represents a score of 3.0 and above, “Partially Reliable” (yellow) represents a score between 2.0 and 3.0, and “Unreliable” (red) represents a score below 2.0.

Authors	Puffing Conditions	Analytical Methods	Derivatization Methods	Other Information	Score and Comments
Auer et al. [53]	√	x	x	x Several flaws	1.0 ⬤
Farsalinos et al. [55]	√	√	√	√	4.0 ⬤
Mallock et al. [57]	√	√	√	√	4.0 ⬤
Uchiyama et al. [49]	√	√	½	√	3.5 ⬤
Salman et al. [58]	½	√	√	x Flawed conclusions on IQOS pollution	2.5 ⬤
Heide et al. [59]	√	√	½ No derivatization	x Poor validation	2.5 ⬤
Wang et al. [60]	√	√	√	x Number of puffs per cigarette not specified	3.0 ⬤
Dusautoir et al. [61]	½	√	√	x HCI regime for sub-ohm devices	2.5 ⬤
Schaller et al. [62]	√	√	√	√	4.0 ⬤
Schaller et al. [45]	√	√	√	√	4.0 ⬤
Jaccard et al. [63]	√	√	√	x No description of conventional cigarettes analyzed	3.0 ⬤
Poynton et al. [64]	√	√	√	√	4.0 ⬤
Buratto et al. [65]	√	√	√	x Number of puffs per cigarette not specified	3.0 ⬤
Crooks et al. [68]	√	√	√	½ Internal methods not available	3.5 ⬤
Eaton et al. [36]	√	√	√	½ Internal methods not available	3.5 ⬤
Forster et al. [69]	√	√	√	½ Internal methods not available	3.5 ⬤
Bentley et al. [46]	√	√	½ No derivatization	√	3.5 ⬤

## Data Availability

Not applicable.

## Abbreviations

°C	Degrees centigrade
BAT	British American Tobacco
CO	Carbon monoxide
CO_2_	Carbon dioxide
CORESTA	Cooperation Centre for Scientific Research Relative to Tobacco
DNPH	2,4-Dinitrophenylhydrazine
EC	Electronic Cigarette
ENDS	Electronic Nicotine Delivery Systems
ETS	Environmental tobacco smoke
FDA	US Food and Drug Administration
FTIR	Fourier Transform Infra-Red Spectrometry
GC-MS	Gas Chromatography–Mass Spectrometry
GC-MS SIM	Gas Chromatography–Mass Spectrometry Selected Ion Monitoring
GVP	Gas vapor phase
HCI	Health Canada Intense
HPHC	Hazardous and Potentially Hazardous Compounds
HPLC-DAD	High-Performance Liquid Chromatography Diode Array Detector
HPLC-UV	High-Performance Liquid Chromatography Ultraviolet
HTP	Heated Tobacco Products
ISO 20778:2018	CORESTA FTC puffing regime (2 s puff, 60 s inter puff, 35 mL puff volume)
ISO 20778:2018	HCI puffing regime (2 s puff, 30 s inter puff, 55 mL puff volume)
ISO	International Organization for Standardization
JTI	Japan Tobacco International
LC-MS/MS	Liquid Chromatography–Mass Spectrometry
MRTP	Modified Risk Tobacco Product
NGDPM	Nicotine-free dry particulate matter
NNK	Tobacco specific nitrosamines
NNN	Tobacco specific nitrosamines
NOx	Nitrogen oxide denoted by “x”
PAHs	Polycyclic aromatic hydrocarbons
PBS	Phosphate-buffered salt solutions
PFBHA	o-(2,3,4,5,6-Pentafluorobenzyl)hydroxylamine
PMI	Phillip Morris International
SPI	TOFMS Single-photon ionization Mass Spectrometry
TGA	Thermogravimetric Analysis
THF	Tetrahydrofuran
THR	Tobacco Harm Reduction
TPM	Total particle matter
TPSAC	Tobacco Product Scientific Advisory Committee
TSNA	Tobacco specific nitrosamines
UHPLC-UV	Ultra High-Performance Liquid Chromatography Ultraviolet
UV	Ultraviolet
VOCs	Volatile organic compounds
WHO	World Health Organization
WS	Smoke-type samples
